# Analyzing Commute Mode Choice Using the LCNL Model in the Post-COVID-19 Era: Evidence from China

**DOI:** 10.3390/ijerph19095076

**Published:** 2022-04-21

**Authors:** Siliang Luan, Qingfang Yang, Zhongtai Jiang, Huxing Zhou, Fanyun Meng

**Affiliations:** 1 School of Transportation, Jilin University, Changchun 130015, China; luansiliang@126.com (S.L.); yangqf@jlu.edu.cn (Q.Y.); zhouhx@jlu.edu.cn (H.Z.); mfy20@mails.jlu.edu.cn (F.M.); 2 Jilin Research Center for Intelligent Transportation System, Changchun 130015, China; 3 Jilin Province Key Laboratory of Road Traffic, Changchun 130015, China

**Keywords:** travel behavior, sustainable modes, COVID-19 pandemic, latent class nested logit model, China

## Abstract

The purpose of this paper is to gain an insight into commuting and travel mode choices in the post-COVID-19 era. The surveys are divided into two waves in Qingdao, China: the first-wave questionnaires were collected under the background of a three-month zero growth of cases; the second wave was implemented after the new confirmed cases of COVID-19. The latent class nested logit (LCNL) model is applied to capture heterogeneous characteristics among the various classes. The results indicate that age, income, household composition, and the frequency of use of travel modes are latent factors that impact users’ attitudes toward mass transit and the private car nests when undergoing the shock of the COVID-19 pandemic. Individuals’ trepidation regarding health risks began to fade, but this is still a vital consideration in terms of mode choice and the purchase of vehicles. Moreover, economic reinvigoration, the increase in car ownership, and an increase in the desire to purchase a car may result in great challenges for urban traffic networks.

## 1. Introduction

The COVID-19 pandemic, one of the most devastating events of the 21st century, has had a dramatically negative impact on regular lifestyles, mobility, travel, transport, and so on [1,2]. There is no doubt that public transit faces great challenges since commuters might choose other modes to avoid the restrictive travel environment. Meanwhile, travel demand has fallen as well; mobility restriction policies and strict lockdowns have directly limited individuals’ social and reactional activities. The world’s governments have taken progressive and unprecedented actions and have achieved a phased victory. People are being vaccinated, aiming to boost immunity. However, the optimistic mood regarding vaccines may be clouded by the appearance of COVID-19 mutations [3]. In other words, the pandemic will not disappear any time soon. There will inevitably be relapses and small-scale outbreaks.

Whether and in what way the pandemic has changed citizens’ travel mode choices, in the long run, is significant for the future development of urban traffic systems. Whether the high degree of trepidation regarding mass transit will have long-term impacts is a topic worth focusing on. Our paper aims to bridge the literature gap between the post-COVID-19 period and mode choice by investigating the causal effect of a small-scale outbreak in Qingdao, China. China has been successful thus far in combatting the pandemic and this has provided valuable information and experiences for other countries in the world. Furthermore, the extra negotiation associated with the intention to make a car purchase and data from the Chinese National Bureau of Statistics assist us in providing an insight into the trends of the development of transportation. The latent class-nested logit (LCNL) model is used to analyze the factors concerned and the characteristics regarding which individuals in each class use their preferred mode, and to capture the attitude heterogeneity in two cross-sectional waves. Our study may also contribute to the literature regarding the application of the LCNL model.

The remainder of the paper is organized as follows. Section 2 presents the literature review of the mobility behavior under the impact of COVID-19 and the discrete choice model we used. Section 3 describes the data collection and method. Section 4 illustrates the results and discusses the LCNL model. The conclusions of our study are presented in Section 5.

## 2. Literature Review

A body of literature on travel mode choice and travel behavior is extensive. According to the current state of knowledge, there are two streams of literature about this matter. The first stream is the effects of COVID-19 on transportation and commute modes. The second stream is the discrete models of travel behavior.

### 2.1. The Effects of COVID-19 on Transportation and Commute Mode

Studies on the onset of the COVID-19 pandemic in connection with mobility choices have recently increased. Although the pandemic has affected the entire world, developed and developing countries with different national conditions, cultural backgrounds, socio-economic characteristics, and anti-pandemic measures may provide some commonalities or different lessons for the global fight against this disruptive disease [4].

A growing number of research studies focus on the impact of COVID-19 on mass transit and the preference changes regarding transportation decision-making. Hung-Hao et al. found that additional confirmed cases of the pandemic decreased underground train use by 1.43% in Taipei due to considerations regarding the infection risk [5]. Labonte LeMoyne et al. investigated 1968 Canadians in early May 2020 and concluded that commuters intend to drive more and use public transport less as a result of the negative impact on health safety, travel experience, and peace of mind regarding mass transit [6]. Campisi et al. carried out an online survey from March to May 2020 in Sicily in Southern Italy [7]. The results suggested that more respondents stated their preference for using micro-mobility instead of public transportation, which was caused by a sense of anxiety. Similar research results are also presented in Jenelius and Cebecauer’s paper [8]. Overall, there was a significant decrease in mass mobility (40–60% across regions) and in Sweden, most of the individuals shifted from public transit to private cars. Beck and Hensher [9,10,11] conducted surveys in two phases in Australia: the first phase was during the initial national outbreak and the second phase was following four to six weeks of relatively few new cases. The results from the first phase show the biggest diminishment in every type of travel mode; aggregate travel rebounded in terms of private car use in the second phase. At the same time, the usage of public transport rebounded as well, but was still far lower than in the pre-COVID-19 era. Przybylowski et al. took the city of Gdansk, Poland, as an example and found that 90% of the respondents would give up or limit the use of mass transit considering the subjective levels of safety and mental comfort, while 75% of them would like to return to public transport if the pandemic situation stabilized [12]. Luan et al. explored the impact of the pandemic on individuals’ travel mode choices in four cities in China and found that people demonstrated regret aversion psychology in travel mode choices to some degree, but the trend became weakened and converged toward the consideration of utility maximization [13]. Samir et al. carried out a nationwide survey during the lockdown in Spring 2020 regarding the willingness to pay for public transport and shared mobility services. The results showed that if the public transport organizations would increase vehicle disinfection rates, travelers were more likely to use public transport during the post-COVID-19 period [14].

Concerning studies on travel behavior and attitudes under the stress of COVID-19, scholars summarized the changes and tendencies of travel mode choices and provided insights for building a “new normal” in a post-pandemic world. As a result of reviewing prior research, the ridership of public transportation had drastic drops and would not recover completely to pre-COVID-19 levels. Some mass transit-oriented users might have seen a permanent shift to other modes such as private cars and micro-mobility. These key findings greatly reflected the short-term effects. However, their prediction regarding the long-lasting impacts may not be apt in high-density populations and heavy-traffic countries such as China. In other words, the background of these studies was that of citizens who were coexisting with the virus; the confirmed cases in their study area did not decline to zero, showing the extent of trepidation regarding mass transit. Every country positively carries out vaccination programs and other prophylactic measures, and the population is becoming vaccinated. We believe that ideal conditions are on their way. Therefore, it is meaningful to explore attitudes toward and preferences for commuting and mobility patterns under this scenario. Nevertheless, the current vaccine program cannot promise to address all the variants, so it is still possible to have a small-scale outbreak at some point. The above-stated condition is a little limited in terms of the previously published literature and current empirical observations, to some extent, are not available in many countries except China. The current paper takes Qingdao, China, as an example and illustrates preferences for travel modes, from the stable zero-growth wave to the wave of new additional local confirmed cases in the post-COVID-19 era, contributing to the existing literature regarding long-term impacts on transportation systems.

### 2.2. Discrete Models of Travel Behavior

Discrete choice models have been widely applied to study the significant determinants impacting travel mode choices and travel behavior [15,16,17]. The results show that socio-demographic characteristics, trip characteristics, geographic setting, and the attributes of various alternatives are vital explanatory factors in individuals’ travel decisions [18,19,20]. Early modeling of the choice of travel mode implemented a one-dimensional approach, such as a multinomial logit model (MNL) [21,22,23], while later researchers developed flexible multi-dimensional logit models to improve the independence of irrelevant alternative (IIA) characteristics of the MNL model, for instance, the nested logit (NL) model [24,25,26], mixed logit (ML) model [27,28,29], and so on. However, the MNL and NL models might be insufficient to uncover the preferences and taste heterogeneity of travelers [30]. The ML model could accommodate random taste heterogeneity, but a prior assumption for the distribution of random parameters is requested and the decision process is encapsulated in a black box, resulting in limited application [31,32,33]. To account for an unobserved heterogeneity with a robust approach, the latent class (LC) model was introduced by Armor in 1968 [34] and was then developed by several researchers for discrete choice analysis [35,36]. It enables researchers to provide sufficient accommodation via a market segmentation method [37,38]. The LC model identifies a finite number of latent classes with heterogeneous characteristics and adopts the specific choice model to estimate individuals’ preferences using homogenous features [39,40]. At present, the LC model has been widely used in many related choice studies, such as the mode decision process [41,42], vehicle ownership [43], residential location [44], willingness-to-pay for vehicles [45], and so on. It was proven that the LC model outperforms the traditional logit models in terms of the goodness of fit [46].

Regarding the specific choice model of the LC model, the LC model could be divided into several types, such as the latent class multinomial logit (LCML) model [47], latent class ordered logit (LCOL) model [48], latent class nested logit (LCNL) model [49], and so on. Most works focus on the LCML; however, fewer researchers use the LCNL model for discrete choice analysis. Since the LCMNL model was developed by the MNL model, it still exhibits IIA properties. Wen et al. used the LCMNL and LCNL models to explore and analyze high-speed rail access mode choices [49]. The results show that the LCNL model overcomes the shortcoming of IIA properties in the MNL model and can estimate parameters more feasibly. Nevertheless, there is limited evidence regarding the empirical analysis of latent factors. Furthermore, Pan also applied the LCNL model to analyze college students’ choice of conventional train trips and high-speed train trips during the Chinese New Year rush [50]. This multi-dimensional choice model garnered good effects and could capture the heterogeneity of the respondents’ preferences. However, this study lacked revealed preference data; hence, it may provide biased results. In our research, we propose an LCNL model to explore the travel preferences and attitudes toward mass transit and auto nests in the post-pandemic era, contributing to the current literature regarding the post-COVID-19 world and the development of the LCNL model.

## 3. Data and Methods

### 3.1. Data

**Figure 1:**
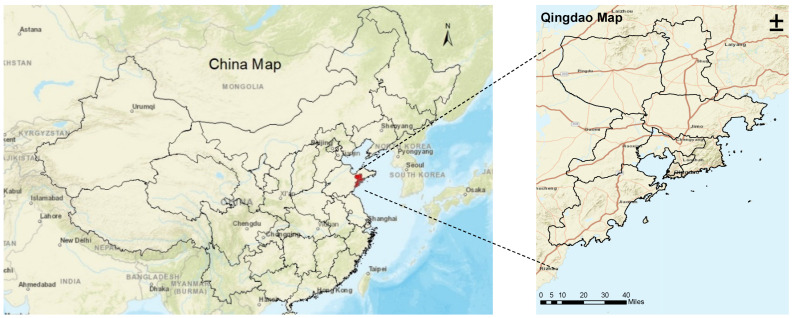
The maps of China and Qingdao.

The data were collected using online surveys via wjx.cn, a platform for the creation and administration of online questionnaires. The study area we selected was Qingdao (also spelled Tsingtao) in China (see Figure 1). Qingdao has the highest GDP of any city in Shandong Province. Since the majority of the population is mainly distributed in the urban city of Qingdao, where transport infrastructures are relatively improved, the survey was conducted in four main regional districts (the Shinan, Shibei, Licang, and Laoshan districts). To ensure regional specificity in our study, the login IP address was restricted to these four districts.

**Figure 2:**
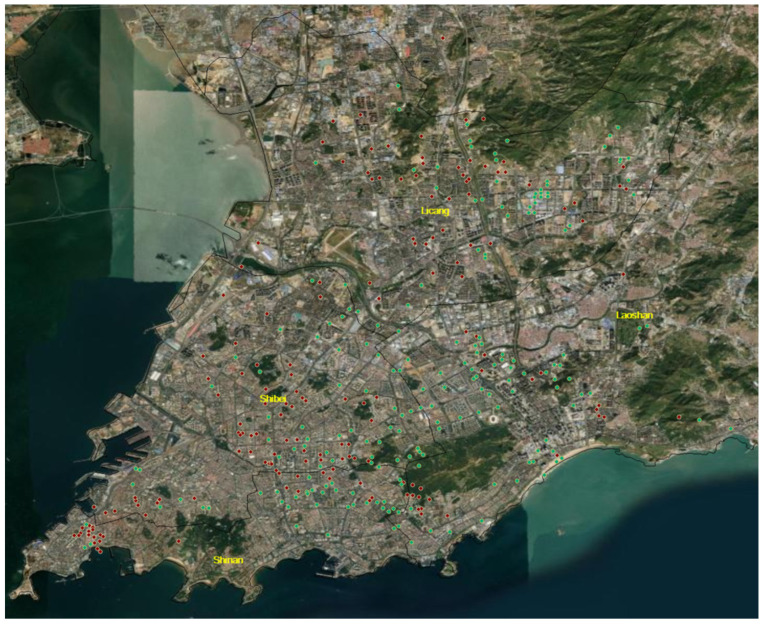
The IP addresses in the two-wave survey.

The main body of our investigation consists of socio-demographic characteristics, trip characteristics, and mode choice preferences, and covers two separate periods. The first-wave online survey was implemented from 1 June 2020 to 20 June 2020, while the second-wave online survey was implemented from 15 October 2020 to 30 October 2020. The reason for selecting these two waves was because the number of coronavirus cases in Qingdao remained stable at zero after 2 March 2020. Commercial activities here have already been got back on the rails, step by step, and citizens have put an end to teleworking and have gradually returned to regular work in the office. Although most of the major gatherings were not allowed and there were still requirements such as wearing a mask in public places, on the whole, people do not have any travel restrictions. Therefore, the first wave was selected in June 2020, after buffering for several months. The time period we selected was a significant point in our study since individuals easily recalled their usual daily routine from the pre-pandemic era, or they formed a new mobility pattern in the post-pandemic era. After all, China was one of the areas hardest hit by the disease; their attitudes toward mass transport and auto transport were heavily impacted by the pandemic. In addition, the second-wave survey was collected after new confirmed cases of COVID-19 were reported on 11 October 2020. At this dividing point, the government declared that Qingdao was changed from a low-risk area to a high-risk area, and people were allowed to travel there only as a last resort. The government organized mass testing for the entire population of roughly nine million people within five days after the discovery of a dozen cases. We adopted this period as our second-phase study time. We would like to scrutinize the effects of the pandemic on motorized transportation in the various “new normal” phases. Even though most of the residents were vaccinated and the government implemented strict measures, it was not true that the virus would no longer be able to spread. The COVID-19 cases may break out in a small-scale area and the numbers may go up again. Divided according to the IP addresses of the respondents, the distribution of the locations of the respondents in the two-wave survey is shown in Figure 2.

**Figure 3:**
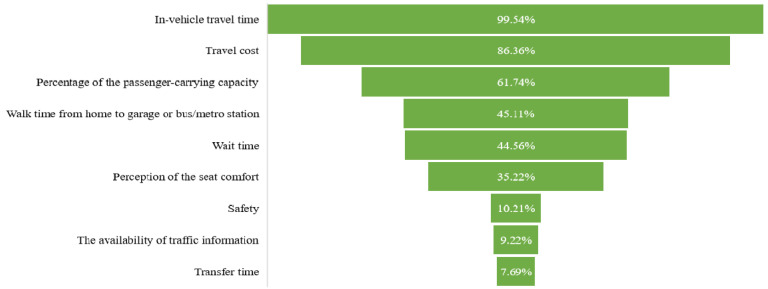
The factors regarding travel mode choice in the pilot survey.

The experiment, complemented by questions associated with revealed preference (RP) and stated preference (SP), was conducted via web-based questionnaires. The RP section included the responders’ socio-demographic and mode preferences, which help explain the variations in the decision process. They established the participants’ gender, age, educational background, monthly income, household composition, car ownership, commuting travel mode, and leisure travel mode. The specific measures are shown in Table 1. All socio-demographics are categorical variables. As for the SP survey, we employed an orthogonal fractional factorial design to investigate the commuting mode choice in a cross-sectional setting. The SP survey presents three hypothetical scenarios based on short (<6 km), medium (6–12 km), and long (>12 km) distances for commuting trips. The alternatives contained public transport (bus and metro) and auto transport (taxi/ride-hailing and private car) nests. We did not provide for active modes (e.g., cycling, e-bikes, bike-sharing, ETW, and walking) as forms of access modes, since we assumed that the distance exceeded the commuters’ acceptable range when using these sustainable modes. Furthermore, inadequate infrastructure and the city’s hilly terrain are also significant barriers to cycling. Before the final questionnaires were completed, we carried out a pilot survey to investigate the timely importance of factors that travelers were concerned about and to select the attributes for our study. As shown in Figure 3, in-vehicle travel time, travel cost, and the percentage of passenger-carrying capacity show an overwhelming superiority among all considerations by the respondents, which are also decisively selected as our attributes. In addition, walk time from home to the garage or bus/metro station and the wait time also have a high share among these factors. To simplify the survey and emphasize the impact of the pandemic on motorized transportation, we integrated walk time and wait time into one variable, namely, out-of-vehicle travel time. It is noteworthy that our two-wave investigations were conducted in spring and autumn when the weather and temperature would be similar in Qingdao. Hence, the attribute of out-of-vehicle travel time disposes of the intervention of bad weather. As for the perception of seat comfort on public transport, Qingdao adopted the uniform standard for bus and metro seats. In addition, it has a lower share of all factors; hence, we do not consider this attribute in our study. Consequently, these hypothetical scenarios for commuters are characterized by in-vehicle travel time, travel cost, out-of-vehicle travel time, and the percentage of passenger-carrying capacity.

The explanatory variables for the discrete choice model are shown in Table 2. The design of the levels of attributes is in accordance with the actual scenarios in Qingdao as far as possible. In terms of the continuous variables of in-vehicle travel time and travel cost, we estimated the results of the linear specification and logarithmic specification and found that the estimation results using the log of travel time and travel cost significantly improved the linear ones in the two waves (we do not show the results of the linear one), and are consistent with the results of [51]. Furthermore, out-of-vehicle travel time for public transport included the walking time from the point of origin to the bus or metro station and the wait time at the bus/metro station, whereas out-of-vehicle travel time for a private car indicates the walking time from home to the garage or parking lot. In terms of taxi/ride-hailing, out-of-vehicle travel time is related to wait time. Therefore, the overall values of the level of out-of-vehicle travel time for the mass transit are lower than those for the auto. As for the percentage of passenger-carrying capacity, PC = 30% denotes an uncrowded ride environment, while PC = 50% illustrates crowded conditions inside mass transport and PC = 80% represents overcrowded conditions.

### 3.2. Method

The latent class model was used to explore discrete preference heterogeneity in terms of travel choice behavior. The benefit of the latent class choice model is that it is not restrictive in terms of observed choice-related attributes and it can exploit the latent heterogeneity, based on variable interactions. Most of the current studies on travel mode choice are based on the MNL specifications in both conditional choice probability and membership probability. However, the choice of MNL model is by definition problematic since there are nested choices in our paper. A nested structure accounts for similarities in the mix of correlation and hierarchy within nests. Therefore, we applied the LCNL model to explore the characteristics of individual travel behavior regarding the onset of COVID-19 during two waves.

The LC model assumes that it can capture individuals’ potential heterogeneous preferences and calibrates those individuals into a finite and fixed number of latent segments. Given that a particular respondent i belongs to segment/class s(s=1,2,…,S), the random utility $U_{im|s}$ of respondent i for the alternative m can be specified as follows [52]:

(1) $$U_{im|s}=V_{im|s}+\varepsilon_{im|s}=W_{mn|s}+Y_{im|s}+\varepsilon_{im|s}$$

where $V_{im|s}$ is the systematic utility and $\varepsilon_{im|s}$ is the random utility. In the NL model, the observed utility $V_{im|s}$ could be divided into two parts: $W_{mk|s}$ and $Y_{im|s}$. $W_{mk|s}$ denotes the constant for all access modes m within a nest n in the class s and $Y_{im|s}$ denotes the utility when the respondent i chooses alternative m in the class s.

**Figure 4:**
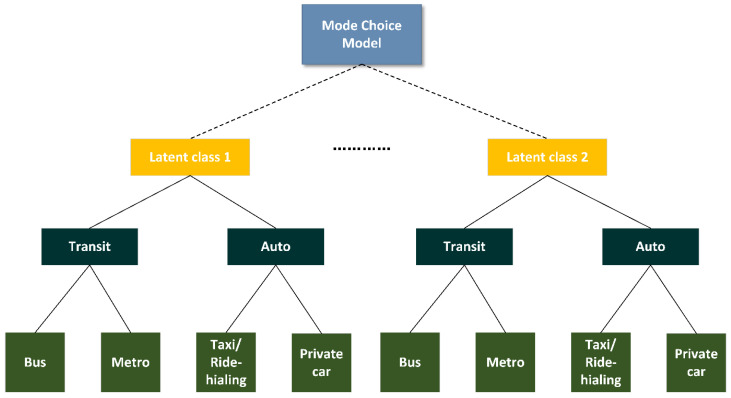
Model structure.

In our paper, it is reasonable to split our choice set into two nests (i.e., mass transit and private auto). As is associated with the specific cases in our study, the random utility function of transit (TR) and auto (AU) for four access modes m can be expressed as:

(2) $$U_{im|s}^{AU}=V_{im|s}^{TR}+\varepsilon_{im|s}^{TR}=W_{m|s}^{TR}+Y_{im|s}^{TR}+\varepsilon_{im|s}^{TR}=\beta_{m,0}^{TR}+\sum_{k}\beta_{k|s}^{TR}x_{imk}^{TR}+\sum_{l}\alpha_{l|s}^{}z_{il}^{}+\varepsilon_{im|s}^{TR}$$

(3) $$U_{im|s}^{AU}=V_{im|s}^{AU}+\varepsilon_{im|s}^{AU}=W_{m|s}^{AU}+Y_{im|s}^{AU}+\varepsilon_{im|s}^{AU}=\beta_{m,0}^{AU}+\sum_{k}\beta_{k|s}^{AU}x_{imk}^{AU}+\sum_{l}\alpha_{l|s}^{}z_{il}^{}+\varepsilon_{im|s}^{AU}$$

where $U_{im|s}^{TR}$ and $U_{im|s}^{AU}$ are the respondent i’s random utility of transit (TR) and auto (AU) for alternative m in the segment s(s=1,2,…S); $V_{im|s}^{TR}$ and $V_{im|s}^{AU}$ represent the systematic utility of transit (TR) and auto (AU), respectively; $W_{m|s}^{TR}$ and $W_{m|s}^{AU}$ represent the parts that are constant for all access modes *m* of the nests of the transit (TR) and auto (AU) in the class s; $Y_{im|s}^{TR}$ and $Y_{im|s}^{AU}$ are the parts that describe the variables of alternative *m* that individual *i* chooses within the nests of the transit (TR) and auto (AU) in the class s; $\varepsilon_{im|s}^{TR}$ and $\varepsilon_{im|s}^{AU}$ represent the random utility of transit (TR) and auto (AU), respectively. The choice transit (TR) and auto (AU) are considered to have segment-specific parameters of $\beta_{k|s}^{TR}$ and $\beta_{k|s}^{AU}$ for attribute *k*, and alternative-specific parameter constants $\beta_{m,0}^{TR}$ and $\beta_{m,0}^{AU}$, respectively. $x_{imk}^{TR}$ and $x_{imk}^{AU}$ are the observable attributes of transit (TR) and auto (AU) for mode m. Subscribe l denotes the individual i’s socio-demographic characteristics; $\alpha_{l|s}^{}$ denotes the estimated parameter of the socio-demographic characteristic l in class s(s=1,2,…,S), while $z_{il}^{}$ denotes the level of individual i’s socio-demographic characteristic l. Figure 4 illustrates our model structure.

The LCNL model comprises two components: the class-membership model and the class-specific model [51]. The class-membership model assigns the membership probability $H_{i}(s)$ to the individual i who belongs to segments s(s=1,2,…S), which can be expressed as follows [53]:

(4) $$H_{i}(s)=\frac{\exp(\sum_{n}\mu_{s}z_{in})}{\sum_{s^{\prime }}\exp(\sum_{n}\mu_{s^{\prime }}z_{in})}$$

where $\mu_{s}$ is the parameters for the segment s, and $z_{in}$ is individual i’s characteristics in the nest n. The membership probability of every segment is determined by the individual’s choice observations and characteristics.

The class-specific model provides the various estimated preferences across each segment. Since the observed utility of the NL model has been divided into two parts, the probability of the LCNL model could also be written as two parts, which is expressed as:

(5) $$P_{i}(m|n,s)\cdot P_{i}(n|s)$$

(6) $$P_{i}(m|n,s)=\frac{\exp(Y_{im|s}/\lambda_{n|s}^{})}{\sum_{m^{\prime }\in N_{n|s}^{}}\exp(Y_{im|s}/\lambda_{n|s}^{})}$$

(7) $$P_{i}(n|s)=\frac{\exp(W_{mn|s}+\lambda_{n|s}\Gamma_{in|s})}{\sum_{n^{\prime }}\exp(W_{mn^{\prime }|s}+\lambda_{n^{\prime }|s}\Gamma_{in^{\prime }|s})}$$

(8) $$\Gamma_{in|s}=\ln\sum_{m^{\prime }}\left[\exp(Y_{im^{\prime }|s}/\lambda_{n|s})\right]$$

where $P_{i}(m|n,s)$ denotes the conditional probability that individual i chooses mode m in the nest n of the class s; $P_{i}(n|s)$; denotes the marginal probability that individual i in the nest n of the class s; $N_{n|s}$ is the set of the nest n in the class s; $\lambda_{n|s}^{}$ is the parameter for the nest n of the class s, and $\Gamma_{in|s}$ denotes the logsum variable in the nest n.

Therefore, the total unconditional probability $P_{i}(m)$ of the LCNL model is given below:

(9) $$P_{i}(m)=\sum_{s}\left[P_{i}(m|n,s)\cdot P_{i}(n|s)]H_{i}(s\right)$$

The Akaike information criterion (AIC) and Bayesian information criterion (BIC) are usually used as measures to determine the optimal number of latent classes, which are expressed by Equations (10) and (11) [54].

(10) $$AIC=-2LL+CK_{\beta }+(C-1)K$$

(11) $$BIC=-2LL+CK_{\beta }+(C-1)K*\ln(N)$$

where LL is the log-likelihood value, solved at the convergence for the estimated parameter; C is the number of latent segments; $K_{\beta }$ is the number of elements in the class-specific model; K is the number of estimated parameters in the classification model; and N is the number of respondents in the sample.

## 4. Results and Discussion

### 4.1. Results

The online survey sample includes 329 valid surveys in wave 1 and 318 valid surveys in wave 2. The profiles of respondents in the two waves are reported in Table 2. The proportion of females (53.33%) in wave 1 is slightly higher than of males (46.67%). However, in wave 2, the proportion of males (56.29%) is higher than of females (43.71%). The age distribution in wave 1 is primarily between 25 and 40 years old and the age distribution is relatively equal in wave 2, except for participants aged over 55. The mean monthly income in wave 1 is higher than in wave 2. People who live in two-generation households are the major respondents in wave 1, accounting for 37.27%, but the household composition of the respondents in wave 2 is couples. A large percentage of respondents had private automobiles, both in wave 1 and wave 2, accounting for 57.58% and 53.77%, respectively. The preference for commuting and entertainment travel modes was primarily by private car in wave 1. In wave 2, the travel mode for work and leisure was more skewed toward mass transit.

In our study, we estimated that the LCNL model with different numbers of the latent class should use three goodness-of-fit measures: log-likelihood, AIC, and BIC. Table 3 shows the cluster results. The log-likelihood function describes the joint probability of the observed data as a function of the parameters of the chosen statistical model. The AIC (Akaike information criterion) and BIC (Bayesian information criterion) are estimators of prediction error and are the criteria for model selection among the different numbers of clusters. As shown in Table 3, every indicator of four classes in wave 1 showed the optimal number of classes. In wave 2, the three-class LCNL model had the lowest AIC value, and the two-class LCNL model had the lowest BIC value. The difference in AIC results between two classes and three classes is not remarkable; however, the BIC result of the two-class LCNL model seems smaller than the three-class model. Therefore, the two-class LCNL model in wave 2 is deemed to give a better result.

- Wave 1

**Figure 5:**
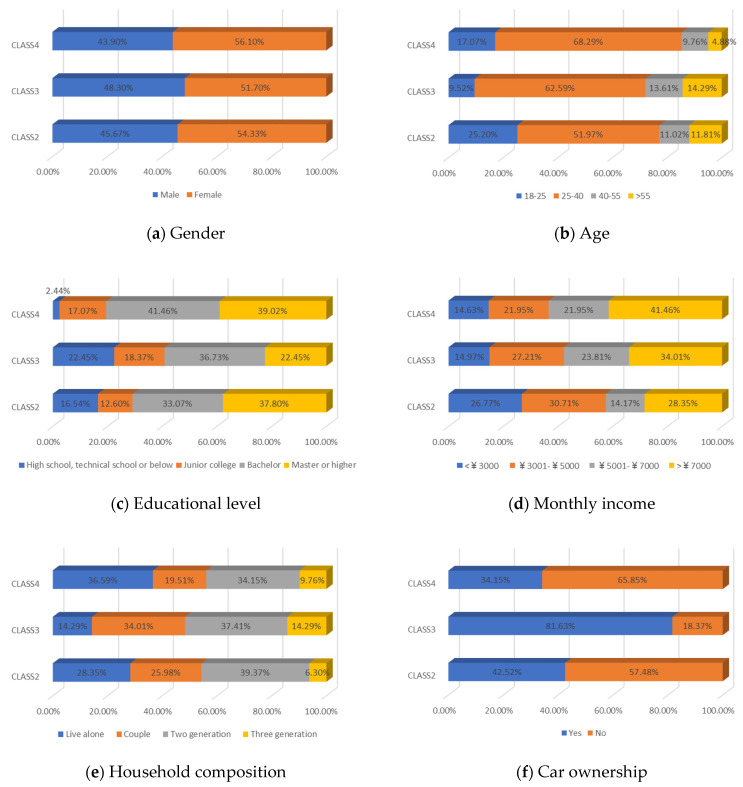

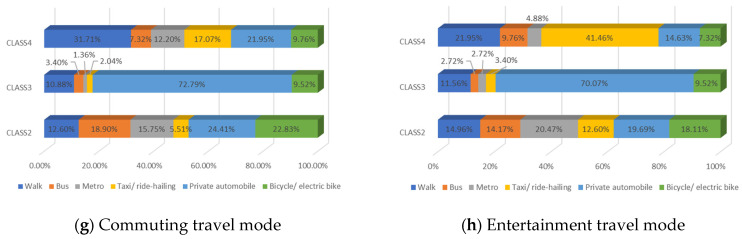
Characteristics of each class.

Table 4 reported the four-class LCNL model in terms of the estimation results of the class-membership model and class-specific model and model statistics (e.g., class size, convergent log-likelihood, and pseudo-R-squared). Class 1 only accounts for 4.26% of the samples, with most of the parameters statistically insignificant at the 90% confidence level. This may illustrate that the respondents in class 1 did not completely understand our choice of tasks. Therefore, we only focus on the remaining classes and analyze them in the following contexts. In our estimation results, classes 2, 3, and 4 contain 38.60%, 44.68%, and 12.46% of the respondents, respectively. The pseudo-R-squared value is 0.2878, which satisfies the requirements [50,54]. Based on the class-membership posterior probabilities from the four-class LCNL model, the proportional distribution of demographic characteristics for each class is shown in Figure 5.

Class 2 is distinct from other classes by the values of age and income. The results provide evidence that the respondents are dominantly female (54.33%) and aged between 25 and 40 (followed by 18–25 years old). The average earnings of the respondents in class 2 are relatively low: low-income (below CNY 3000) and middle-income (CNY 3001–5000) individuals in the samples account for 26.77% and 30.71%, respectively. Meanwhile, the highest proportion of respondents received a master’s degree or higher education (37.80%), followed by a bachelor’s degree (33.07%). The majority of the respondents live with children or parents (39.37%) or are couples (25.98%). The largest percentage of respondents in this class do not have a private automobile (57.48%). As for their daily travel mode, micro-mobility, public transit, and autos account for 35.43%, 34.65%, and 29.92%, respectively, in terms of work. Micro-mobility, public transit, and auto travel occupy 33.07%, 34.64%, and 32.29% respectively, in terms of recreational activities. In the class-specific model, all the estimated parameters are significant at a 90% confidence level. The coefficient estimates for in-vehicle travel time and travel cost are negative, which is consistent with our expectations. In terms of in-vehicle travel time, the influence of public transit is higher than for an auto. Respondents would show stronger negative emotions with the increase in travel time for mass transit. However, the respondents are more sensitive to the variable of travel cost in the auto travel mode. This is understandable since individuals prefer lower fares and less in-vehicle travel time; the characteristics of mass transport are lower in cost but higher in in-vehicle travel time; conversely, the characteristics of the automobile are higher in cost but less in in-vehicle travel time. Similar trends are also shown in class 3 and class 4. As for the out-of-vehicle travel time, the respondents in class 1 represent the aversion when they wait beyond 10 min. The results of the percentage of passenger-carrying capacity (PC_TR_) show that the negative emotions would gather pace as the crowd increased in public transit.

The third class represents individuals who are primarily female (51.70%); most of them are aged between 25 and 40 years old (62.59%) and are above 55 years old (14.29%). The distribution of educational levels is comparatively balanced and the majority of them achieved bachelor’s degrees (36.73%). The monthly income of the respondents in class 3 is high, and high-income (>CNY 7000) individuals account for 34.01% of respondents, followed by middle- and high-earning individuals (CNY 5001–7000, 23.81%). People living in a two-generation household and living with a couple are a higher proportion, accounting for 37.41% and 34.01%, respectively. The distinctive characteristic of this class is the proportion of car ownership, where 81.63% of the respondents have private automobiles. Therefore, it is easy to understand that these individuals would like to drive for work or leisure, occupying 72.79% and 70.07%, respectively. A similar tendency to in-vehicle travel time and travel cost could be seen with class 2. However, the main difference is the attitude toward out-of-vehicle travel time and the percentage of passenger-carrying capacity in terms of mass transport. As car-dominated users, they have a lower tolerance for the crowded situation of mass transit. Hence, the signs of the parameters of “PC = 50%” and “PC = 80%” negatively illustrate their anxiety regarding travel and the riding environment. Moreover, they are sensitive to longer out-of-vehicle travel time and they regard a 10-minute wait time as their scope of acceptance. This may also be associated with their favorite commuting mode since the distance from home to the parking lot/garage is generally no more than ten minutes.

In terms of class 4, most of the class are also female (56.10%) and are aged between 25 and 40 (68.29%). Bachelor’s and higher education levels account for 41.46% and 39.02%, respectively, which means that the individuals in class 4 have excellent educational backgrounds. The largest percentage (41.46%) of the respondents may obtain above CNY 7000 in income every month. We observed that 36.59% of the respondents lived alone and 34.15% of them lived with parents or children. Most of them do not own a private car (65.85%). In addition, 31.71% of the respondents walk to work, which represents the highest proportion of commuting travel modes. This may be because their workplace is close to their home. The members of class 4 would like to wave down taxis or call ride-hailing services to leisure places, which mirrors living standards and a concept of consumption for this high-income population. As for the variables of in-vehicle travel time and travel cost, the similarity of their attitude is also shown in class 4. The estimated parameters of OTT_TR_ = 10 min and OTT_AU_ = 10 min represent the various perceptions. It is understandable that out-of-vehicle travel time for mass transit indeed takes a longer time than the auto; hence, a 10-minute out-of-vehicle travel time for the different modes has a dissimilar worth in terms of criteria, based on the travelers’ cognition. In terms of the unique attributes of mass transport (PC_TR_), the respondents are apt to choose the transit method where the percentage of the passenger-carrying capacity is less than 50%—this is reasonable since the ride sensation and social distancing make the traveler feel comfortable and safe.

- Wave 2

The background of wave 2 was after additional and new confirmed cases of COVID-19 and all dwellers were tested in Qingdao. Based on the results in Table 3, the samples can be clustered into two classes. Class 1 and class 2 contain 60.69% and 39.31% respondents of the sample population, respectively. The estimation results of the latent class analysis in wave 2 are shown in Table 5 and the characteristics of every class are illustrated in Table 6.

In class 1, the majority of the respondents are male (55.44%) and in terms of age are between 40 and 55 years old (followed by 25–40 years old). The educational levels of these individuals are mainly high school, technical school or below, and junior college, with a share of 31.09% and 37.82%, respectively. The income characteristics of the travelers in class 1 are middle- and high-level. The highest proportion of the population lives with a couple (41.45%). Furthermore, this group of individuals mostly do own a car (72.02%), but they usually travel by mass transit for work (46.12%) and leisure (37.82%). The estimation of the class-specific model shows that all parameters are statistically significant at a 95% confidence level. As for the in-vehicle travel time of the mass transit nest and auto nest, the influence of public transport is much smaller than it is for cars, whereas the parameter value of travel cost of the auto nest is relatively smaller. Interestingly, the attitude of respondents regarding in-vehicle travel time and travel cost exceeded our expectations and it reflects the opposite trend compared with wave 1. The members in class 1 have a higher tolerance for longer out-of-vehicle travel time for public transport since they would like to choose sustainable travel modes for daily mobility patterns and they may be accustomed to the characteristics of the public modes, even if they do own private cars. The coefficient estimations of the percentage of passenger-carrying capacity are consistent with our expectations. In general, individuals prefer low congested conditions.

In terms of class 2 in wave 2, members are mostly male (57.60%), young (the share of age between 18 and 25 years old and 26–40 years old are 30.40% and 36.80%), better educated (35.20% junior college and 29.60% Bachelor), middle income (34.40% of them earn between CNY 3001 and 5000 monthly, while 33.60% of them earn between 5001 and CNY 7000), live with more than two or three family members (34.40% of them live with their couples and 47.20% live in two- or three-generation families). In class 2, the majority of the respondents do not have private cars (74.40%). The respondents in the second class commute by mass transit (46.12%), by car or taxi (26.43%), and on foot or by bike (27.46%). However, most of them (46.40%) have a personal preference for taking the bus for leisure activities. These class members show a similar trend for the variables of in-vehicle travel times and travel cost with class 1. In addition, when the out-of-vehicle travel time exceeds 10 min, the individuals in class 2 show strong adverse emotion. They also feel extremely uncomfortable with the overcrowded conditions of mass transit.

### 4.2. Discussion

In this subsection, we will discuss the results in Section 4.1 from the perspective of comparison between the two waves. Meanwhile, we will combine the rest of the investigation results from the questionnaires (e.g., the intentions regarding car purchase and the considered factor of mode choice) and willingness-to-pay (WTP) in our survey to analyze the mode choice in the post-pandemic era.

- Comparison within Two Waves

In wave 1, regarding age and class, older people are more likely to be associated with class 3 and class 4, whereas younger people are more prevalent in class 2. Furthermore, the education parameters show that more highly educated individuals are presented in the population in class 2 and class 4, compared with class 3. Monthly income and the number of household members of the individuals impact the travel modes in daily life. Respondents with middle and high incomes reflect a strong association with class 3 and class 4, whereas respondents with middle- and low-earning capacity illustrate a better fit with class 2. Individuals who live in a three-generation family are more associated with class 4, and individuals who live with parents, children, or as couples are more prevalent in class 2. Individuals in class 3 are more likely to own private automobiles. According to the results of their daily mobility pattern, the members in class 3 also have strong preferences regarding driving for commuting and leisure activities. The use of public transport is more associated with class 2 than class 4. According to the significant estimated results and the characteristics of “younger, low-income, high-education, carless and mass transit-oriented”, we infer that the respondents in class 2 may be university students or graduates, and label them as “students or graduates from the university”. The characteristics of “older, normal-educated, middle- and high-income and car-dominated” in the respondents in class 3 made us name this group “car-oriented users”. Meanwhile, class 4 reflects individuals with the characteristics of high income, are highly educated, living with more family members, and show low car ownership, being labeled as “high-income and large-household carless individuals”.

Concerning the attitude of the auto nest, the class 3 “car-oriented users” in wave 1 manifest a higher negative emotion regarding long in-vehicle and out-of-vehicle travel time and the higher travel cost of the automobile mode, compared with class 2 and class 4. Meanwhile, the aversion sensation regarding the crowded riding environment of mass transport is valued least here among the three classes. The attitude toward “OTT_TR_ = 10 min” also differs here from the other two classes. This might be because car-dominated users have a higher sensitivity toward their usual travel mode; therefore, their perception of every attribute of the automobile is more intense. Nevertheless, their negative attitude toward the congested conditions of public transport is not remarkable. In a nutshell, despite the shock of the COVID-19 pandemic, the individuals in class 3 will not change their commute mode compared with before the pandemic; hence, they will not show an enormous aversion to the use of mass transit.

**Figure 6:**
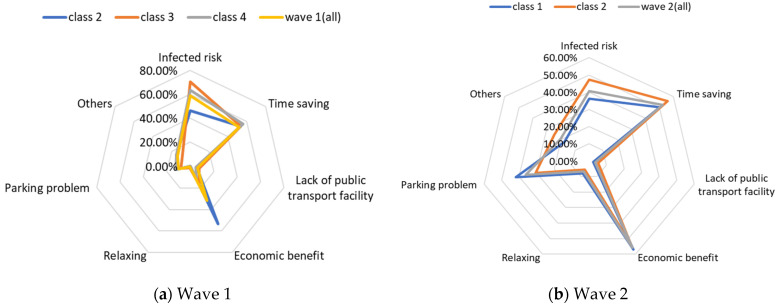
Factors representing the consideration of the two waves in terms of mode choice.

Class 2, “students or graduates from the university” in our study, has some overlap with class 4, “high-income and large-household carless individuals”, regarding the proportion of higher-educated respondents, car ownership, and public transport-oriented users. Since the average income level of class 2 is the lowest among the three classes, the aversion emotion to high travel costs for the auto and transit nest is the most intense compared with the others. Furthermore, the use frequency of public transport in class 2 is more frequent than for class 4. Therefore, the respondents in class 2 are more sensitive to in-vehicle and out-of-vehicle travel time in terms of mass transit than in class 4, whereas they reflect a lower negative perception regarding longer travel time for the auto mode. As for the percentage of passenger-carrying capacity, when the onboard environment is in a crowded condition, the members of class 2 show an optimistic attitude, while the individuals of class 4 feel pessimistic. The discrepancy of PC = 50% between class 2 and class 4 may be caused by family concerns. The individuals in class 2 are more likely to live alone and they will not pay much attention to the risk of infecting their housemates. Our questionnaires were also linked with the factors considered when the respondents made travel choice decisions, as shown in Figure 6. The loading of the factor of infection risk in class 2 also verifies the above conclusion. In a word, income level, household composition, and the frequency of use of their travel mode would significantly become potential factors that influenced travel mode choices in the first phase, during the onset of the COVID-19 pandemic.

In wave 2, the corresponding parameters of gender show that when the passenger was a female, she was more likely to belong to class 2. Individuals aged between 25 and 55 years old in class 1 had a higher probability of being female, whereas older women had a strong likelihood of falling into class 2. The educational level in class 1 was lower than in class 2 and the number of family members in class 1 was fewer than in class 2. The individual who lived in a household of three-generation was more likely to belong to class 2. Moreover, people who held private vehicles were more commonly associated with class 1. Thus, it can be concluded that class 1 can be classified as “young car-owners” and class 2 can be labeled as “carless people who live with more family members”.

In the class-specific model, we can find the estimated parameters of in-vehicle travel time and travel cost regarding mass transit, and the automobile nest in class 1 is smaller than in class 2. This result can be interpreted as showing that the socio-demographic characteristics (younger and a higher income) of class 1 cause a stronger response regarding fluctuating fares and time. Meanwhile, most of them owned private cars, which means that if they did not enjoy the experience of their current travel mode, they would consider choosing another mode to replace it. This would also explain the divergence of the sign of OTT_AU_ = 10 min and PC_AU_ = 50%. Combined with the results in Figure 6, class 2, “carless people who live with more family members”, would be more inclined to consider the risk of infecting family members. However, most of them are car-free and may not have other substitutes for work trips, so they are accustomed to the features of mass transit during peak hours. It is not hard to explain why they are living with several generations and why they might not be able to afford to relax their vigilance in terms of health risks, but they have a positive attitude regarding the crowded conditions of public transport (PC_AU_ = 50%). In wave 2, we found that age, household composition, and the use frequency of their travel mode are vital latent factors that impact their attitude toward various modes.

- Comparison between Two Waves

A total of five latent classes during two waves of the COVID-19 pandemic have been identified using the LCNL model. The investigated populations are different and random during the two waves; hence, it is not reasonable to compare the estimated values between the two waves. Therefore, we utilized the willingness-to-pay (WTP) factor to provide an illuminating insight into the changes and characteristics among the above segments of respondents during the two waves. In Table 7, we illustrate the WTP estimation results of two waves for each class, taken from the LCNL model results. The target of WTP estimation is to analyze how much utility is gained or lost when the attributes change and to discover the monetary sensitivity of every class [55]. Furthermore, besides the analysis of WTP and the estimated results, the results of the investigation of the extra considered factors in Figure 6 and the individuals’ car-purchase desires are also covered in this part.

Several observations can be made in relation to Table 7 regarding the WTP of the two waves. In light of the issue of public transport, the overall WTP for each attribute indicates that the respondents in wave 1 would be willing to pay more for a better riding environment. As in the above-estimated results, the attitude of respondents toward two nests reflects the opposite trends between the two waves. The members in wave 1 show a stronger negative emotion regarding longer travel time and more congested conditions of mass transit. Combined with the considered factors in Figure 6, the higher share of individuals in wave 1 would like to take the infection crisis into account, while the lower proportion of individuals in wave 1 would consider the economic benefits. We found that since the respondents in wave 1 undervalued economic factors and perceived a major virus risk on board, they were willing to spend more money on a better service on mass transit to guarantee their safety.

Initially, we assumed that individuals might be wary of the virus risk to some extent and would prefer to pay more, especially after several confirmed cases in wave 2. However, the results are not consistent with our expectations. This might be because people in China had only just suffered the catastrophe and social and economic activities were reinvigorating gradually; hence, these individuals had a lingering fear of the pandemic. Since COVID-19 is an acute respiratory tract infection, the difficulty of keeping physical social distancing is a major crisis in mass transit. There is no doubt that a crowded riding environment would motivate the travelers’ psychological anxiety and panic, which could mainly embody the variable of the percentage of the passenger-carrying capacity of the transit. In addition, in-vehicle travel time also plays a vital role in travel mode choices under the impact of the COVID-19 pandemic. The interior space of the mass transport is relatively confined and stuffy, causing a greater risk of infection over time. As for the potential crisis regarding the long out-of-vehicle travel time of the mass mobility mode, bus stops and metro stations are significant centralized sites and it is also difficult to keep safe social distancing during peak hours. The longer the wait time, the higher the risk of infection. Based on an analysis of the influences of the above-mentioned variables and the extra considered factors in Figure 6, we could observe that although the pandemic has been brought under control, the consideration of infection still occupies a high share factor for travel mode choices. Therefore, individuals are willing to pay more for safer riding environments in the short term. However, the trend of infection consideration and willingness-to-pay for public transport is remarkably weakened; people are more likely to take time-saving and economic factors into account in the long run, even if the pandemic had a local small-scale relapse.

In light of the auto nests in wave 1, the overall values of the WTP are smaller than in the mass transit nest. It is possible that people deem the auto modes to be relatively safer than mass transit and they would not be prepared to pay more for a better experience of riding cars. However, the total tendency of WTP for the auto mode in wave 2 differs from wave 1. Individuals are eager to improve the service quality of private vehicles or carpools. Furthermore, most of the commuters in wave 2 are public transport-oriented users, so they are willing to occasionally pay more to enjoy a better journey experience by auto.

**Figure 7:**
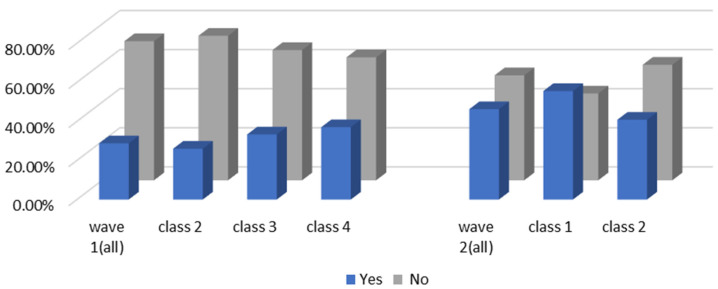
Car purchase intention of the carless cohort in two waves.

**Figure 8:**
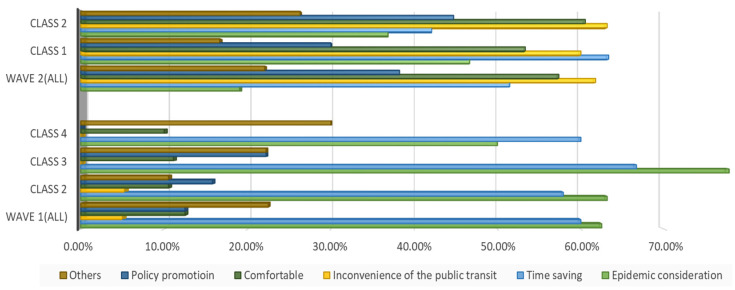
The reasons for purchasing a car.

Besides their WTP, negotiation of the intention of car purchase also provides evidence to help us explore the impact of COVID-19 on travel mode choices. Figure 7 and Figure 8 report the car purchase intention and the reasons for buying a car in the two waves, respectively. In general, people worrying about the infection risk may have intensely excited a greater intention of car purchase in wave 1. Nevertheless, the income of residents experienced negative growth in the first quarter of 2020, being affected by the pandemic. Data from the Chinese National Bureau of Statistics show that the national per capita disposable income of residents in the first quarter of 2020 was CNY 8561. With the recovery of economic growth, residents’ income has also shown an improvement trend and finally ushers in positive growth in the third quarter of 2020. The data also show that in the first three quarters of 2020, the national per capita disposable income of residents was CNY 23,781, a nominal increase of 3.9% year-on-year. Combined with the reasons for car purchase in Figure 8, despite public health concerns as the most remarkable factors, poor financial background and lower disposable income levels would discourage the desire for automobile purchase. By contrast, an economic resurgence in wave 2 may become one of the outstanding prerequisites for car purchase. Furthermore, we observed that individuals in wave 2 would like to make a synthesis of their demand. Undoubtedly, time-saving needs and the inconvenience of public transport are the top two factors of all the cohort’s reasons. Still, pandemic considerations are a vital factor but have lower shares in resolving them.

Moreover, we would like to explore other interesting findings recorded after several new confirmed COVID-19 cases. As mentioned above, 53.77% of the respondents had private cars as a whole, with car owners accounting for 72.02% in class 1 in wave 2. In total, 46.26% of the carless respondents in wave 2 were eager to purchase cars. Data from the Chinese Ministry of Public Security show that the number of motor vehicles in China reached 372 million in 2020, which is an increase of 24 million compared to 2019, a year-on-year increase of 6.90%. In 2019, there was an increase of 21 million vehicles from 2018, a year-on-year increase of 6.4%. Furthermore, Chinese economic growth reports represented that the GDP for the whole year of 2019 increased by 6.1% over the previous year. The economic growth rate in 2020 was about 2.3%. In 2019, the growth rates of vehicle ownership and GDP were almost synchronous. However, compared with the amplification of the GDP, the increase in vehicle ownership was larger in 2020. Therefore, we infer that the pandemic did, indeed, promote the desire for private cars in carless households to some extent. The carless cohort considered that it was necessary to keep a private car in the face of the outbreak of the pandemic or the restriction of public transport at the height of the pandemic. It seems that the urban traffic system will meet a great challenge in the future, with the growing number of private cars. Nevertheless, unlike the high-frequency use of the private car among class 3 “car-oriented users” in wave 1, most of the individuals of class 1 in wave 2 owned their own private automobiles but they were still willing to use mass transit for their work trips. Here, 10.03% of the respondents would consider parking problems concerning their commute in wave 1, but 36.08% of these individuals might emphasize parking issues in wave 2. This difference may be caused by economic activity, the condition of the automobile population, and the individuals’ travel willingness. A more active trade economy and the increasing amount of travel by car resulted in high parking fees and a shortage of parking spaces. Besides the parking issues, the traffic jam and economic benefits also play significant roles in mode choice, helping us to understand why the car owners in wave 2 did not drive for commuting trips. Consequently, even though the number of vehicles was growing by leaps and bounds, traffic issues would be coming along soon as well. Traffic conditions may be in need of dynamic balancing in the long run; however, mass transit is still the main transport method for commuters, as before.

## 5. Conclusions and Future Research

In our paper, we explored the characteristics of commuting mode choice, using RP and SP data in two progressive waves of the post-COVID-19 pandemic in Qingdao, China. During the first wave, Qingdao recorded zero cases in three months of the pandemic and people went back to normal working from home patterns. Schools also reopened and economic activities were markedly undertaken. The second wave was selected after a dozen new confirmed cases; all the dwellers in Qingdao were tested. We adopted the LCNL model to classify three classes in wave 1: students or graduates from the university, car-oriented users, and high-income and large-household carless individuals. Two classes were found in wave 2: young car-owners and carless people who lived with several family members. The attitude and analysis of each class are also provided as a contribution to the literature on travel mode choices in the post-COVID-19 era. In a nutshell, there are several preliminary findings:

1. Age, income, household composition, and the frequency of use of travel modes are significant latent factors that impact the respondents’ attitude toward the mass transit nest and the auto nest under the impact of the COVID-19 pandemic. In wave 1, car owners were almost all car-oriented users and they showed less anxiety regarding public transport since they did not change their daily mobility pattern and the virus crisis from mass transit would not significantly affect their travel mode choice. Younger, middle- and low-income respondents living in a household with fewer family members, and public transport-dominated users might overlook the health risks on board and show less aversion emotion regarding mass transit. In wave 2, car owners may not be car-oriented commuters and they are more sensitive to both transit and auto nests, since they have substitutes for their current mode. Carless individuals usually take mass transit for work trips and keep a more positive attitude toward public transport, which is different from that in other developing countries [56]. In line with the reported findings in previous studies [57], older adults living in a big family are inclined to pay more attention to the risk of the infection, while it is inadequate to change their commute mode.
2. Against the backdrop of controlling the spread of the disease, individuals’ trepidation regarding the infection risk gradually faded, but it was still a critical consideration in terms of travel mode choice. More individuals would prefer to take the health issue into account for commute mobility patterns and they are willing to pay more to improve the mass transit service in wave 1 than wave 2.
3. The economic factor is a foundational base for the intention of car purchase. The pandemic enables researchers to stimulate the desire of buying a vehicle to some extent, but this is not the uppermost consideration in wave 2. On the contrary, due to the impact of the pandemic on the national economy and employment market, the individuals in wave 1 may not show a great demand for private cars.
4. In light of economic reinvigoration and the increase in car ownership, urban traffic is faced with a great challenge but still remains in dynamic equilibrium. Since a large number of citizens are willing to take public transport to go to work even if they are car owners, mass transit is still the mainstream mode for commuters in the post-COVID-19 era.

The firm confidence in public transport in post-pandemic times is at the root of the government’s strict prevention strategies. Comprehensive information dissemination regarding policy and digital infrastructure plays a significant role in mitigating the spread of the pandemic. The public receives the timely broadcasting of information and adjusts their travel behavior and psychological expectations. Meanwhile, applying a digital infrastructure could help individuals to replace the face-to-face experience to some extent. Individuals are mandated to wear face masks in public areas and are required to maintain social distancing as far as possible. Working from home, online education, online meetings, health codes, customized bus routes, and an intelligent logistic service provide assistance for post-pandemic recovery. Besides the success of combating the domestic spread of the virus, the majority of new confirmed cases are from international arrivals. Therefore, the Chinese border policy is to require all new arrivals from abroad to show negative reports of the PCR- and IgM antibody blood tests and to undergo centralized isolation for two weeks. However, these strict regulations may not avoid new outbreaks. Once there are new additional confirmed cases of COVID-19, all local citizens must conduct a nucleic acid test within a short period. By following these policies, the trepidation and anxiety of the public regarding the crisis of infection rates would abate dramatically and citizens would return to normal life immediately.

Some limitations of this study need further research. Firstly, due to random surveys, respondents with different socio-demographic variables do not supply similar background information in the two waves. It is unreasonable to use the latent transition method [58] or a longitudinal study [59] to analyze direct changes in the same group in the two waves. Therefore, we cannot make a specific comparison between the five classes of the two waves. If the survey is conducted for the same population and the two-wave survey time is closer, we might anticipate changes in mobility choices with the onset of an important event. Furthermore, there are some omitted factors, such as seat comfort, transfer time, etc., which would impact the estimated results. In addition, we only provide using the bus, metro, taxi/ride-hailing, and a private car as alternatives in the SP survey because the questionnaires are based on three different distance scenarios and we tacitly assumed that people would not select micro-mobility for a median- or long-distance commute. Finally, we did not draw enough conclusions regarding the use of taxis or ride-hailing; it is considered an alternative for individuals who are not able to afford to run their own private car but we did not obtain enough evidence to summarize the characteristics of carpooling in the post-pandemic era. Further research should consider the above-mentioned issues and improve our survey. In addition, it is hypothesized that the findings reported here are based on Chinese society. We would like to explore other countries with similar socio-demographic and economic characteristics if their citizens have similar travel preferences and travel mode choices. The different prevention measures in these countries could have various impacts on travel behavior.

## Figures and Tables

**Table 1:** Model variables.

Attributes	Code	Description	Levels	
In-vehicle travel time (TR)	Log(ITT)_TR_	In-vehicle travel time using the transit mode	Scenario 1	20, 30, 40 (min)
			Scenario 2	30, 40, 50 (min)
			Scenario 3	45, 55, 65 (min)
Travel cost (TR)	Log(TC)_TR_	Travel cost for the transit mode	Scenario 1	Bus: 1, 2 (CNY) Metro: 2, 3 (CNY)
			Scenario 2	Bus: 1, 2 Metro: 3, 5 (CNY)
			Scenario 3	Bus: 1, 2 Metro: 4, 6 (CNY)
Out-of-vehicle travel time (TR)	OTT_TR_	Out-of-vehicle travel time of the transit includes walking time from the origin to the bus or metro station and wait time at the bus/metro station	-	5, 10, 15, 20 (min)
In-vehicle travel time (AU)	Log(ITT)_AU_	In-vehicle travel time using the auto mode	Scenario 1	10, 15, 20 (min)
			Scenario 2	15, 20, 25 (min)
			Scenario 3	30, 35, 40 (min)
Travel cost (AU)	Log(TC)_AU_	Travel time for the auto mode	Scenario 1	15, 20, 25 (CNY)
			Scenario 2	20, 25, 30 (CNY)
			Scenario 3	35, 40, 45 (CNY)
Out-of-vehicle travel time (AU)	OTT_AU_	Out-of-vehicle travel time of the transit includes walking time from the origin to the garage or parking lot, or wait time for taxi/ride-hailing	-	2, 6, 10, 14 (min)
Percentage of the passenger-carrying capacity (PC)	PC_TR_	Percentage of the passenger-carrying capacity of the transit mode	-	30%, 50%, 80%

**Table 2:** Descriptive statistics of the socio-demographic variables.

Variables	Category	Wave 1	Wave 2
Gender	Male	46.67%	56.29%
	Female	53.33%	43.71%
Age	18–25	17.27%	23.90%
	25–40	57.58%	34.91%
	40–55	12.73%	31.13%
	>55	12.42%	10.06%
Educational level	High school, technical school, or below	18.79%	27.99%
	Junior college	16.06%	36.79%
	Bachelor’s degree	35.45%	26.10%
	Master’s degree or higher	29.70%	9.12%
Monthly income (CNY)	<¥3000	20.61%	10.69%
	¥3001–¥5000	28.18%	26.42%
	¥5001–¥7000	19.39%	34.59%
	>¥7000	31.82%	28.30%
Household composition	Live alone	23.64%	21.70%
	Couple	28.79%	38.68%
	Two generations	37.27%	24.53%
	Three generations	10.30%	15.09%
Car ownership	Yes	57.58%	53.77%
	No	42.42%	46.23%
Commute travel mode	Walk	15.76%	18.87%
	Bus	10.61%	23.58%
	Metro	8.18%	22.64%
	Taxi/ride-hailing	5.15%	14.47%
	Private automobile	45.15%	12.58%
	Bicycle/electric bike	15.15%	7.86%
Entertainment travel mode	Walk	16.06%	22.64%
	Bus	9.09%	29.87%
	Metro	10.00%	13.52%
	Taxi/ride-hailing	11.52%	12.58%
	Private automobile	40.91%	9.12%
	Bicycle/electric bike	12.42%	12.26%

Notes: CNY (¥) is the Chinese currency unit. CNY 1 = USD 0.1547 = EUR 0.1279 in January 2021.

**Table 3:** Information criteria for the number of latent classes.

Classes	WAVE 1				WAVE 2			
	Number of Parameters	Log-Likelihood	AIC	BIC	Number of Parameters	Log-Likelihood	AIC	BIC
2	43	−5049.00	10,184.02	10,471.63	43	−7768.90	15,623.81	15,909.86
3	86	−4619.90	9393.83	9908.84	86	−7728.10	15,610.11	16,122.34
4	123	−4281.10	8784.25	9526.67	123	−7729.43	15,703.66	16,442.08
5	145	−4355.15	8932.56	9768.15	145	−7738.40	15,766.71	16,731.31
6	179	−4708.01	9214.015	10,276.27	179	−7737.10	15,832.11	17,022.89
7	213	−4722.46	9678.926	10,461.59	213	−7742.40	15,910.89	17,327.85

**Table 4:** Estimation results of the latent class analysis in Wave 1.

Parameters	Class 1		Class 2		Class 3		Class 4	
Class-Membership Model	Value	t-Stat.	Value	t-Stat.	Value	t-Stat.	Value	t-Stat.
ASC_Class			3.883	16.128	3.236	12.867	2.483	9.907
Male			0.227	2.334	0.219	2.231	0.161	1.500
Female			−0.227		−0.219		−0.161	
Age (18–25)			0.366	1.752	−0.028	−0.130	−0.204	−0.850
Age (25–40)			0.234	2.397	0.399	2.382	0.247	1.347
Age (40–55)			−0.813	−4.652	−0.940	−5.288	−0.357	−1.756
Age (>55)			0.214		0.568		0.314	
Education (High school, technical school, or below)			−1.012	−5.466	−0.476	−2.547	−2.120	−8.410
Education (Junior college)			0.108	0.599	0.190	1.085	0.642	3.143
Education (Bachelor’s)			−0.758	−4.639	−1.048	−6.351	−0.236	−2.303
Education (Master’s or higher)			1.662		1.334		1.715	
Income (<3000)			−0.708	−4.765	−0.989	−6.414	−1.142	−6.527
Income (3001–5000)			0.186	1.262	−0.062	−0.414	−0.288	−1.773
Income (5001–7000)			0.521	2.628	0.933	4.671	1.159	5.523
Income (>7000)			0.002		0.118		0.270	
Household (live alone)			−0.606	−3.283	−0.705	−3.787	−0.735	−3.682
Household (couple)			0.789	4.566	0.568	3.251	0.293	1.530
Household (two generations)			0.670	4.358	0.484	3.100	0.415	2.490
Household (three generations)			−0.852		−0.346		0.027	
Car ownership (Yes)			−0.488	4.977	0.899	6.705	−0.668	3.359
Car ownership (No)			0.488		−0.899		0.668	
Commute mode (Walk)			−0.879	−3.829	−0.378	−1.599	0.215	0.893
Commute mode (Bus)			0.988	1.591	−0.652	−2.191	−0.908	−2.943
Commute mode (Metro)			1.001	2.739	0.665	1.064	1.427	2.427
Commute mode (Taxi/ride-hailing)			0.417	1.287	−0.928	−1.042	2.332	1.730
Commute mode (Private car)			−2.341	−2.692	3.128	2.973	−0.658	−2.253
Commute mode (Bike/electric bike)			0.814		−1.835		−2.408	
Entertainment mode (Walk)			−2.101	−7.775	−0.378	−9.093	0.615	−8.276
Entertainment mode (Bus)			1.029	−4.422	−0.652	−6.328	−0.508	−2.931
Entertainment mode (Metro)			1.034	−0.123	−0.665	−3.785	1.427	−4.625
Entertainment mode (Taxi/ride-hailing)			1.536	3.818	−0.928	−3.230	1.332	6.442
Entertainment mode (Private car)			−2.366	−1.333	4.258	3.128	−0.458	−2.248
Entertainment mode (Bike/electric bike)			0.868		−1.635		−2.408	
Class-specific model								
Constant (metro)	0.035	0.023	0.662	4.003	−0.073	−3.193	0.725	2.088
Constant (taxi/ride-hailing)	−0.842	0.085	−0.887	5.125	−2.545	3.312	1.101	1.988
Constant (private car)	0.745	1.243	−0.161	−2.112	2.575	3.112	−0.249	2.105
Log(ITT)_AU_	−1.249	−0.850	−0.114	−5.525	−0.117	−3.047	−0.091	−2.249
Log(ITT)_TR_	0.155	−0.977	−0.141	−4.971	−0.166	−2.100	−1.190	−2.460
Log(TC)_AU_	−1.246	−1.882	−0.127	−1.825	−0.141	−3.110	−0.119	−2.246
Log(TC)_TR_	−0.120	−0.770	−0.107	−1.961	−0.093	−2.984	−0.080	−2.418
OTT_TR_ = 5	0.369	0.864	0.327	2.484	1.538	1.862	0.283	3.186
OTT_TR_ = 10	0.004	1.569	0.144	1.982	−0.237	1.977	0.095	1.874
OTT_TR_ = 15	−0.310	−1.255	−0.122	2.103	−0.485	−2.107	−0.105	−2.362
OTT_TR_ = 20	−0.464		−0.350		−0.815		−0.273	
OTT_AU_ = 2	0.441		0.711	−3.516	1.517	5.141	0.583	1.987
OTT_AU_ = 6	−0.115		−0.219	1.968	0.980	3.121	0.148	2.336
OTT_AU_ = 10	−0.142		−0.228	−2.361	−0.672	−3.100	−0.336	−3.155
OTT_AU_ = 14	−0.184		−0.264		−1.824		−0.394	
PC_TR_ = 30%	0.160	0.366	0.575	2.001	0.388	1.644	0.553	1.743
PC_TR_ = 50%	−0.035	−0.156	0.034	2.211	−0.058	1.821	−0.081	1.781
PC_TR_ = 80%	−0.125		−0.608		−0.332		−0.472	
Model statistics								
Class size	4.26%		38.60%		44.68%		12.46%	
Number of observations	5934							
Covergent log-likelihood	−4281.1							
Pseudo R-squared	0.2878							

**Table 5:** Estimation results of the latent class analysis in Wave 2.

Parameters	Class 1		Class 2	
Class-Membership Model	Value	t-Stat.	Value	t-Stat.
ASC_Class			−0.1827	−1.984
Male			−0.253	−2.991
Female			0.253	
Age (18–25)			0.002	4.759
Age (25–40)			−0.027	−2.276
Age (40–55)			−0.330	−2.504
Age (>55)			0.354	
Education (High school, technical school, or below)			−0.617	−4.003
Education (Junior college)			−0.150	−0.464
Education (Bachelor)			−0.010	−0.057
Education (Master’s or higher)			0.778	
Income (<3000)			−0.410	−1.168
Income (3001–5000)			0.406	0.386
Income (5001–7000)			−0.045	1.977
Income (>7000)			0.049	
Household (live alone)			−0.218	−1.963
Household (couple)			−0.213	−2.643
Household (Two generations)			−0.391	1.751
Household (Three generations)			0.822	
Car ownership (Yes)			−0.754	−2.134
Car ownership (No)			0.754	
Commute mode (Walk)			−0.052	−1.042
Commute mode (Bus)			−0.212	−3.163
Commute mode (Metro)			0.387	−1.758
Commute mode (Taxi/ride-hailing)			0.308	0.016
Commute mode (Private car)			−0.229	0.444
Commute mode (Bike/electric bike)			−0.202	
Entertainment mode (Walk)			0.196	0.687
Entertainment mode (Bus)			0.752	1.025
Entertainment mode (Metro)			−0.185	−2.874
Entertainment mode (Taxi/ride-hailing)			−0.172	0.534
Entertainment mode (Private car)			−0.328	−2.387
Entertainment mode (Bike/electric bike)			−0.263	
Class-specific model				
Constant (metro)	0.235	2.679	0.384	2.986
Constant (taxi/ride-hailing)	0.283	1.969	−0.004	1.961
Constant (private car)	−0.374	−2.652	−0.053	2.661
Log(ITT)_AU_	−0.435	−1.987	−0.131	−2.512
Log(ITT)_TR_	−0.271	−2.330	−0.025	3.174
Log(TC)_AU_	−0.329	−2.089	−0.260	−2.016
Log(TC)_TR_	−1.945	−1.981	−0.701	−2.025
OTT_TR_ =5	2.079	2.661	2.190	−1.841
OTT_TR_ = 10	0.162	2.256	0.801	−1.996
OTT_TR_ = 15	0.046	2.455	−1.490	−2.103
OTT_TR_ = 20	−2.286		−1.501	
OTT_AU_ = 2	0.522	1.997	1.844	3.254
OTT_AU_ = 6	0.426	2.164	0.624	3.111
OTT_AU_ = 10	−0.340	−3.127	0.919	2.630
OTT_AU_ = 14	−0.608		−3.387	
PC_TR_ = 30%	1.411	2.365	0.942	2.001
PC_TR_ = 50%	−0.504	−3.682	0.138	2.211
PC_TR_ = 80%	−0.907		−1.081	
Model statistics				
Class size	60.69%		39.31%	
Number of observations	5724			
Convergent log-likelihood	−7768.9			
Pseudo R-squared	0.267			

**Table 6:** Characteristics of each class in Wave 2.

	Gender	Age		Educational Level
Class 1	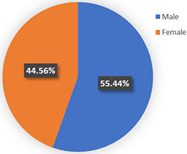	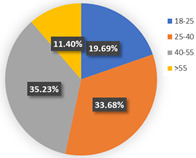		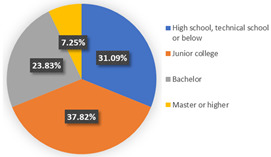
Class 2	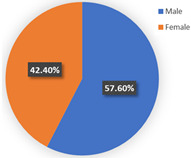	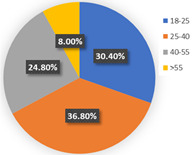		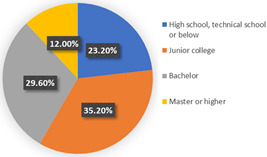
	Car ownership	Income		Household composition
Class 1	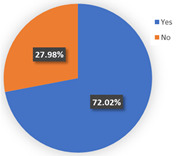	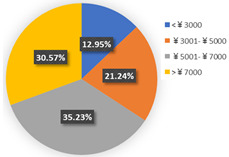		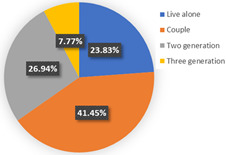
Class 2	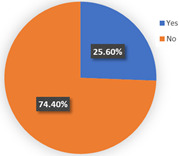	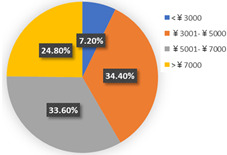		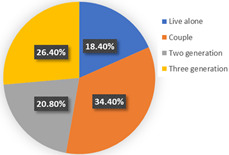
	Commute travel mode		Entertainment travel mode	
Class 1	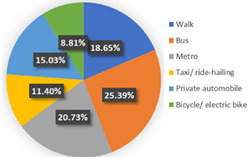		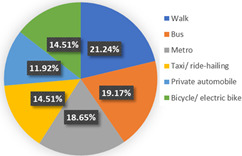	
Class 2	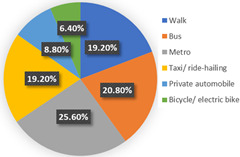		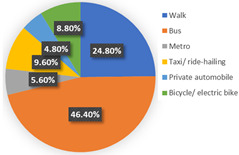	

**Table 7:** Willingness to pay for each of the classes.

	Wave 1			Wave 2	
Transit	Class 2	Class 3	Class 4	Class 1	Class 2
ITT	−1.318	−1.785	−2.388	−0.139	−0.036
PC = 30%	5.374	4.172	6.913	0.725	1.344
PC = 50%	0.318	−0.624	−1.013	−0.259	0.197
PC = 80%	−5.682	−3.570	−5.900	−0.466	−1.542
OTT = 5 min	3.056	16.538	3.538	1.069	3.124
OTT = 10 min	1.346	−2.548	1.188	0.083	1.143
OTT = 15 min	−1.140	−5.215	−1.313	0.024	−2.126
OTT = 20 min	−3.271	3.516	−3.413	−1.175	−2.141
Auto	Class 2	Class 3	Class 4	Class 1	Class 2
ITT	−0.898	−0.830	−0.765	−1.322	−0.504
OTT = 2 min	5.598	10.759	4.899	1.587	7.092
OTT = 6 min	−1.724	6.950	1.244	1.295	2.400
OTT = 10 min	−1.795	−4.766	−2.824	−1.033	3.535
OTT = 14 min	−2.079	−12.936	−3.311	−1.848	−13.027

## Data Availability

The data that support the findings of this study are available from Jilin University but restrictions apply to the availability of these data, which were used under license for the current study, and so are not publicly available. Data are however available from the authors upon reasonable request and with permission of Jilin University.

## References

[1] Cucinotta D., Vanelli M. (2020). WHO Declares COVID-19 a Pandemic. Acta Bio Med. Atenei Parm..

[2] Afifi R.A., Novak N., Gilbert P.A., Pauly B., Abdulrahim S., Rashid S.F., Ortega F., Ferrand R.A. (2020). ‘Most at risk’ for COVID19? The imperative to expand the definition from biological to social factors for equity. Prev. Med..

[3] Sumathi S., Swathi K., Suganya K., Sudha B., Poornima A., Hamsa D., Banupriya S.K. (2021). A broad perspective on COVID-19: A global pandemic and a focus on preventive medicine. Tradit. Med. Res..

[4] Shaw R., Kim Y.-K., Hua J. (2020). Governance, technology and citizen behavior in pandemic: Lessons from COVID-19 in East Asia. Prog. Disaster Sci..

[5] Chang H.-H., Lee B., Yang F.-A., Liou Y.-Y. (2021). Does COVID-19 affect metro use in Taipei?. J. Transp. Geogr..

[6] Labonte-LeMoyne E., Chen S.L., Coursaris C.K., Senecal S., Leger P.M. (2020). The Unintended Consequences of COVID-19 Mitigation Measures on Mass Transit and Car Use. Sustainability.

[7] Campisi T., Basbas S., Skoufas A., Akgun N., Ticali D., Tesoriere G. (2020). The Impact of COVID-19 Pandemic on the Resilience of Sustainable Mobility in Sicily. Sustainability.

[8] Jenelius E., Cebecauer M. (2020). Impacts of COVID-19 on public transport ridership in Sweden: Analysis of ticket validations, sales and passenger counts. Transp. Res. Interdiscip. Perspect..

[9] Beck M.J., Hensher D.A. (2020). Insights into the impact of COVID-19 on household travel and activities in Australia-The early days of easing restrictions. Transp. Policy.

[10] Beck M.J., Hensher D.A. (2020). Insights into the impact of COVID-19 on household travel and activities in Australia-The early days under restrictions. Transp. Policy.

[11] Beck M.J., Hensher D.A., Wei E. (2020). Slowly coming out of COVID-19 restrictions in Australia: Implications for working from home and commuting trips by car and public transport. J. Transp. Geogr..

[12] Przybylowski A., Stelmak S., Suchanek M. (2021). Mobility Behaviour in View of the Impact of the COVID-19 Pandemic-Public Transport Users in Gdansk Case Study. Sustainability.

[13] Luan S., Yang Q., Jiang Z., Wang W. (2021). Exploring the impact of COVID-19 on individual’s travel mode choice in China. Transp. Policy.

[14] Awad-Núñez S., Julio R., Gomez J., Moya-Gómez B., González J.S. (2021). Post-COVID-19 travel behaviour patterns: Impact on the willingness to pay of users of public transport and shared mobility services in Spain. Eur. Transp. Res. Rev..

[15] Sobieniak J., Westin R., Rosapep T., Shin T. (1979). Choice of access mode to intercity terminals. Transp. Res. Rec..

[16] Garling T., Gillholm R., Garling A. (1998). Reintroducing attitude theory in travel behavior research-The validity of an interactive interview procedure to predict car use. Transportation.

[17] Hensher D.A., Rose J.M., Rose J.M., Greene W.H. (2005). Applied Choice Analysis A Primer.

[18] Rashidi T.H., Mohammadian A. (2011). Household travel attributes transferability analysis: Application of a hierarchical rule based approach. Transportation.

[19] Raveau S., Guo Z., Muñoz J.C., Wilson N.H.M. (2014). A behavioural comparison of route choice on metro networks: Time, transfers, crowding, topology and socio-demographics. Transp. Res. Part A Policy Pract..

[20] Westin K., Jansson J., Nordlund A. (2018). The importance of socio-demographic characteristics, geographic setting, and attitudes for adoption of electric vehicles in Sweden. Travel Behav. Soc..

[21] Bhat C.R. (1998). Analysis of travel mode and departure time choice for urban shopping trips. Transp. Res. Part B Methodol..

[22] Bhat C.R. (2000). Incorporating Observed and Unobserved Heterogeneity in Urban Work Travel Mode Choice Modeling. Transp. Sci..

[23] Lin J.-J., Yu T.-P. (2011). Built environment effects on leisure travel for children: Trip generation and travel mode. Transp. Policy.

[24] Forinash C.V., Koppelman F.S. (1993). Application and interpretation of nested logit models of intercity mode choice. Transp. Res. Rec..

[25] Heiss F. (2002). Structural Choice Analysis with Nested Logit Models. Stata J..

[26] Lee B.H.Y., Waddell P. (2010). Residential mobility and location choice: A nested logit model with sampling of alternatives. Transportation.

[27] Hess S., Bierlaire M., Polak J.W. (2005). Estimation of value of travel-time savings using mixed logit models. Transp. Res. Part A Policy Pract..

[28] Hensher D.A., Rose J.M., Greene W.H. (2008). Combining RP and SP data: Biases in using the nested logit ‘trick’–contrasts with flexible mixed logit incorporating panel and scale effects. J. Transp. Geogr..

[29] Leite Mariante G., Ma T.-Y., van Acker V. (2018). Modeling discretionary activity location choice using detour factors and sampling of alternatives for mixed logit models. J. Transp. Geogr..

[30] Chamberlain G. (1980). Analysis of covariance with qualitative data. Rev. Econ. Stud..

[31] Walker J., Ben-Akiva M. (2002). Generalized random utility model. Math. Soc. Sci..

[32] Fosgerau M. (2006). Investigating the distribution of the value of travel time savings. Transp. Res. Part B Methodol..

[33] Fu X. (2020). How habit moderates the commute mode decision process: Integration of the theory of planned behavior and latent class choice model. Transportation.

[34] Armor D.J. (1969). Latent Structure Analysis. Paul, F. Lazarsfeld, Neil, W. Henry. Am. J. Sociol..

[35] Kamakura W.A., Russell G.J. (1989). A Probabilistic Choice Model for Market-Segmentation and Elasticity Structure. J. Mark. Res..

[36] Greene W.H., Hensher D.A. (2003). A latent class model for discrete choice analysis: Contrasts with mixed logit. Transp. Res. Part B Methodol..

[37] Lee B.J., Fujiwara A., Zhang J., Sugie Y. Analysis of mode choice behaviours based on latent class models. Proceedings of the 10th International Conference on Travel Behaviour Research.

[38] Zhou H., Norman R., Xia J.H., Hughes B., Kelobonye K., Nikolova G., Falkmer T. (2020). Analysing travel mode and airline choice using latent class modelling: A case study in Western Australia. Transp. Res. Part A Policy Pract..

[39] Boxall P.C., Adamowicz W.L. (2002). Understanding Heterogeneous Preferences in Random Utility Models: A Latent Class Approach. Environ. Resour. Econ..

[40] Shen J. (2009). Latent class model or mixed logit model? A comparison by transport mode choice data. Appl. Econ..

[41] Prato C.G., Halldorsdottir K., Nielsen O.A. (2017). Latent lifestyle and mode choice decisions when travelling short distances. Transportation.

[42] Ton D., Zomer L.B., Schneider F., Hoogendoorn-Lanser S., Duives D., Cats O., Hoogendoorn S. (2020). Latent classes of daily mobility patterns: The relationship with attitudes towards modes. Transportation.

[43] Kim S.H., Mokhtarian P.L. (2018). Taste heterogeneity as an alternative form of endogeneity bias: Investigating the attitude-moderated effects of built environment and socio-demographics on vehicle ownership using latent class modeling. Transp. Res. Part A Policy Pract..

[44] Lee Y., Circella G., Mokhtarian P.L., Guhathakurta S. (2019). Heterogeneous residential preferences among millennials and members of generation X in California: A latent-class approach. Transp. Res. Part D Transp. Environ..

[45] Ferguson M., Mohamed M., Higgins C.D., Abotalebi E., Kanaroglou P. (2018). How open are Canadian households to electric vehicles? A national latent class choice analysis with willingness-to-pay and metropolitan characterization. Transp. Res. Part D Transp. Environ..

[46] Anowar S., Yasmin S., Eluru N., Miranda-Moreno L.F. (2014). Analyzing car ownership in Quebec City: A comparison of traditional and latent class ordered and unordered models. Transportation.

[47] Liu Z.Y., Kemperman A., Timmermans H., Yang D.F. (2021). Heterogeneity in physical activity participation of older adults: A latent class analysis. J. Transp. Geogr..

[48] Shannon D., Murphy F., Mullins M., Rizzi L. (2020). Exploring the role of delta-V in influencing occupant injury severities—A mediation analysis approach to motor vehicle collisions. Accid. Anal. Prev..

[49] Wen C.-H., Wang W.-C., Fu C. (2012). Latent class nested logit model for analyzing high-speed rail access mode choice. Transp. Res. Part E Logist. Transp. Rev..

[50] Pan X.F. (2021). Investigating college students’ choice of train trips for homecoming during the Spring Festival travel rush in China: Results from a stated preference approach. Transp. Lett..

[51] Seelhorst M., Liu Y. (2015). Latent air travel preferences: Understanding the role of frequent flyer programs on itinerary choice. Transp. Res. Part A Policy Pract..

[52] Train K. (2003). Discrete Choice Methods with Simulation.

[53] Hensher D.A., Greene W.H. (2002). Specification and estimation of the nested logit model: Alternative normalisations. Transp. Res. Part B Methodol..

[54] Louviere J.J., Hensher D.A., Swait J.D. (2000). Stated Choice Methods: Analysis and Applications.

[55] Boto-Garcia D., Mariel P., Pino J.B., Alvarez A. (2020). Tourists’ Willingness to Pay for holiday trip characteristics: A Discrete Choice Experiment. Tour. Econ..

[56] Abdullah M., Ali N., Hussain S.A., Aslam A.B., Javid M.A. (2021). Measuring changes in travel behavior pattern due to COVID-19 in a developing country: A case study of Pakistan. Transp. Policy.

[57] Meng Y., Khan A., Bibi S., Wu H., Lee Y., Chen W. (2021). The Effects of COVID-19 Risk Perception on Travel Intention: Evidence From Chinese Travelers. Front. Psychol..

[58] Bray B.C., Dziak J.J. (2018). Commentary on latent class, latent profile, and latent transition analysis for characterizing individual differences in learning. Learn. Individ. Differ..

[59] Kim J., Kwan M.-P. (2021). The impact of the COVID-19 pandemic on people’s mobility: A longitudinal study of the U.S. from March to September of 2020. J. Transp. Geogr..

